# The Hospital World

**Published:** 1911-09-16

**Authors:** 


### THE HOSPITAL WORLD.

### Death of a Medical J.P.

Dr. Edwin Dean, J.P., has died at Slaithwaite, Huddersfield, in his seventieth year. Dr. Dean had always taken a prominent part in the public work of the locality, where, two centuries ago, his great-grandfather established a practice, and in 1906 he was placed on the Commission of Peace for the West Riding of Yorkshire. For many years all medical practice in the Colne Valley was practically monopolised by the Dean family and their assistants. Dr. Edwin Dean studied at Manchester and Glasgow, and qualified in 1869 as a licentiate of the Society of Apothecaries. He held the appointments of public vaccinator and district medical officer to the Huddersfield Union, and was certifying factory surgeon for the neighbourhood. Dr. Dean was the author of a treatise on "Mind and Brain."

### Death of a Bristol Practitioner and Crimean Veteran.

Dr. Henry Robert Dew, who has just died in Somerset, came of a family having long and historical associations in that county. The Dew family have had in their possession for many generations a manse at Coleford which tradition relates once afforded shelter to Charles I., while an ancestor is said to have saved the king's life with the aid of a battleaxe still preserved among the treasures of the family. Dr. Dew's grandfather was a well-known practitioner in Bristol, and he himself entered the profession at an early age, for he served in the Crimean War as staff assistant surgeon to Lord Raglan. He was born in 1834, and took his medical studies at St. George's Hospital, London. After the war he settled in Bristol, where he enjoyed a successful practice for a very long period.

### Charitable Contributions of the Railway Companies.

According to a White Paper issued this week it appears that during last year the sum of £16,255 was contributed by the railway companies of the United Kingdom to charitable institutions and associations of various character, not directly controlled by the companies and not founded for the exclusive benefit of the companies' servants. Of that sum the English and Welsh companies contributed £13,812, the Irish companies £1,557, and the Scottish companies £885. This figure is interesting when compared with the percentage of sums contributed by other companies. It would be instructive, and we venture to think practically helpful as well, to compile a statistical record showing the percentage which various dividend-paying concerns contribute yearly to charities. We imagine that the results would very likely surprise many people who hold that capital and charity are almost diametrically opposed interests.

### A Suffolk Medical Family.

The death is announced of Dr. George Edwards Jeaffreson, of Framlingham, Suffolk, at the age of seventy-six. Dr. Jeaffreson belonged to a medical family long established in Suffolk, his father (William Jeaffreson, F.R.C.S.) having practised in Framlingham, where Dr. G. Cordy Jeaffreson now carries on the practice. The late Dr. G. E. Jeaffreson took an active interest in the affairs of the British Medical Association, and was a former President of the East Anglia branch, continuing his connection as vice-president up to the time of his death. He was also a Justice of the Peace for the county of Suffolk. Dr. Jeaffreson pursued his medical studies in London at University College, becoming qualified in 1857 as a member of the Royal College of Surgeons. He contributed papers on rural hygiene and on the relation of insanity to alcoholism.

